# Artificial Neural Network Modeling to Predict Neonatal Metabolic Bone Disease in the Prenatal and Postnatal Periods

**DOI:** 10.1001/jamanetworkopen.2022.51849

**Published:** 2023-01-23

**Authors:** Honglin Jiang, Jialin Guo, Jing Li, Chunlin Li, Wenchong Du, Federico Canavese, Charlie Baker, Hao Ying, Jing Hua

**Affiliations:** 1 Department of Mother and Children's Health Care, Shanghai First Maternity and Infant Hospital, Tongji University School of Medicine, Shanghai, China; 2 Department of Epidemiology, School of Public Health, Fudan University, Shanghai, China; 3 Department of Psychology, Nottingham Trent University, Nottingham, United Kingdom; 4 Department of Pediatric Orthopedic Surgery, Lille University Hospital and Faculty of Medicine, Lille, France; 5 Faculty of Medicine, Jeanne de Flandre Hospital, Rue Eugène Avinée, Lille, France

## Abstract

**Importance:**

Early recognition of metabolic bone disease (MBD) in infants is necessary but difficult; an appropriate tool to screen infants at risk of developing MBD is needed.

**Objectives:**

To develop a predictive model for neonates at risk for MBD in the prenatal and postnatal periods and detect the pivotal exposed factors in each period.

**Design, Setting, and Participants:**

A diagnostic study was conducted from January 1, 2012, to December 31, 2021, in Shanghai, China. A total of 10 801 pregnant women (singleton pregnancy, followed up until 1 month after parturition) and their infants (n = 10 801) were included. An artificial neural network (ANN) framework was used to build 5 predictive models with different exposures from prenatal to postnatal periods. The receiver operating characteristic curve was used to evaluate the model performance. The importance of each feature was examined and ranked.

**Results:**

Of the 10 801 Chinese women who participated in the study (mean [SD] age, 29.7 [3.9] years), 7104 (65.8%) were local residents, 1001 (9.3%) had uterine scarring, and 138 (1.3%) gave birth to an infant with MBD. Among the 5 ANN models, model 1 (significant prenatal and postnatal factors) showed the highest AUC of 0.981 (95% CI, 0.970-0.992), followed by model 5 (postnatal factors; AUC, 0.977; 95% CI, 0.966-0.988), model 4 (all prenatal factors; AUC, 0.850; 95% CI, 0.785-0.915), model 3 (gestational complications or comorbidities and medication use; AUC, 0.808; 95% CI, 0.726-0.891), and model 2 (maternal nutritional conditions; AUC, 0.647; 95% CI, 0.571-0.723). Birth weight, maternal age at pregnancy, and neonatal disorders (anemia, respiratory distress syndrome, and septicemia) were the most important model 1 characteristics for predicting infants at risk of MBD; among these characteristics, extremely low birth weight (importance, 50.5%) was the most powerful factor. The use of magnesium sulfate during pregnancy (model 4: importance, 21.2%) was the most significant predictor of MBD risk in the prenatal period.

**Conclusions and Relevance:**

In this diagnostic study, ANN appeared to be a simple and efficient tool for identifying neonates at risk for MBD. Combining prenatal and postnatal factors or using postnatal exposures alone provided the most precise prediction. Extremely low birth weight was the most significant predictive factor, whereas magnesium sulfate use during pregnancy could be an important bellwether for MBD before delivery.

## Key Points

**Question:**

Can the artificial neural network (ANN) predict risk for neonatal metabolic bone disease (MBD) in the prenatal and postnatal periods?

**Findings:**

In this diagnostic study of 10 801 participants, among 5 ANN models with different exposures, the models including significant prenatal and postnatal factors and postnatal factors alone had the best ability to predict infants at risk for MBD.

**Meaning:**

These findings suggest that the ANN, using prenatal and postnatal factors, could be an efficient tool for identifying neonates at risk for BMD.

## Teaser

This diagnostic study explores the optimal model for predicting neonatal metabolic bone disease.

## Introduction

Metabolic bone disease (MBD), a bone health disorder characterized by hypophosphatemia, hyperphosphatemia, and skeletal demineralization, is commonly observed in preterm infants who are deprived of fetal mineral accumulation.^1,2,3^ The incidence of MBD is related to birth weight and ranges from 16% to 40% in very low-birth-weight (VLBW, <1500 g) and extremely low-birth-weight (ELBW, <1000 g) infants.^1^

Metabolic bone disease may be due to both prenatal and postnatal factors that cause disturbances in calcium and phosphorus metabolism, such as maternal nutrient availability, placental insufficiency, drugs that induce bone resorption, parenteral nutrition, and neonatal disorders.^2,3,4^ Skeletal manifestations of MBD include osteopenia, osteoporosis, rickets, and pathologic fractures in severe cases. In addition to a subsequent increase in bone fragility, MBD can also impair both long- and short-term longitudinal growth of long bones.^5,6^ The negative effect on bone mineralization may delay somatic development and increase susceptibility to other chronic diseases in adulthood.^7^

Screening of infants at risk of developing MBD is necessary, although diagnostic methods vary widely in different countries and institutions.^8,9^ In addition, the late onset of clinical symptoms and signs combined with the lack of specific biochemical markers make early recognition of MBD challenging.^3,10^ Although numerous epidemiologic studies^7,11,12,13^ have identified several risk factors for MBD during the neonatal period, little work has evaluated the prediction of their development. In addition, traditional risk prediction models are limited in forecasting the probability of an outcome,^14^ especially a multifactorial disease with a context of large, high-dimensional, and imbalanced data set.^15^ However, they cannot quantify the importance of each feature on predicting the outcomes.^16^ Furthermore, contemporary research often investigates preterm infants, using relatively small sample sizes,^17^ with few having studied MBD among infants in general.^18,19^

Because clinical data acquired during the time frame covered by a research period may not include all relevant risk factors, a set of accurate predictive tools for MBD applicable to different scenarios is needed. In recent years, the artificial neural network (ANN) technique, a highly flexible and accurate machine learning algorithm, has been widely used for diagnostic and prognostic prediction of various diseases.^20,21^ An ANN simulates the behavior of biological neurons to learn and model the nonlinear relationships between variables and allow computers to predict new data using the learned potential patterns.^22^ The output value is compared with that expected output. Learning proceeds by modifying the weight of the connections between neurons until a minimum error of the network is reached. After training, the ANN can generate outputs (prediction) on a new data set based on the accumulated knowledge. In some cases, ANN might provide a more accurate estimation than conventional approaches.^23^

We aimed to develop serial ANN models for the risk of MBD based on the hypothesis that exposures spanning the antenatal to postpartum periods that influence fetal bone formation might indicate infants at higher risk for MBD. The primary objective was to identify the optimal model for prediction; the secondary objective was to detect the pivotal exposed factors in each period by examining the impacts of each model’s features.

## Methods

### Study Design and Participants

After securing institutional review board approval from the Shanghai First Maternity and Infant Hospital and the Tongji University School of Medicine, a diagnostic study was conducted in a cohort of pregnant Chinese women from January 1, 2012, to December 31, 2021. All participants gave written informed consent before enrollment and the data collected from them were deidentified. Participants were recruited early in pregnancy and followed up until 1 month after parturition with clinical data recorded at each visit. This study followed the Transparent Reporting of a Multivariable Prediction Model for Individual Prognosis or Diagnosis (TRIPOD) reporting guideline.^24^

Inclusion criteria were as follows: (1) singleton pregnancy; (2) complete clinical data during the antenatal, delivery, and postpartum periods; and (3) surviving infants with detailed values of alkaline phosphatase. Metabolic bone disease was diagnosed as a peak serum alkaline phosphatase level greater than 500 U/L (to convert to microkatals per liter, multiply by 0.0167)^18^ 72 hours after birth measured by the 2-amino-2-methyl-1-propanol method. Women who gave birth to an infant with MBD were selected as the case group.

Detailed calculation of the required sample size for this study is described in the eMethods in Supplement 1. We excluded 2559 participants with twin or triplet pregnancy and 3158 with missing data from any variables of interest. A total of 10 801 women and their infants were finally included in the analyses (eFigure 1 in Supplement 1).

### Data Collection and Handling

We collected maternal and neonatal characteristics data from electronic health records for analysis (eTable 1 in Supplement 1). Characteristics included the following: (1) demographic data and previous pregnancy history: age at pregnancy, occupation, ethnicity (on resident’s identification card), region, prepregnancy body mass index (BMI), parity, and uterine scarring; (2) nutritional conditions during pregnancy: anemia, deficiencies of folic acid, ferritin, and vitamin D, and supplementation of corresponding nutrients (folic acid, iron, calcium, and vitamin D); (3) complications and comorbidities: placenta previa, placental abruption, gestational diabetes, gestational hypertension, kidney disease, and fever; (4) medication use during pregnancy: dexamethasone, magnesium sulfate, antibiotics, and furosemide; (5) birth outcomes: prematurity, sex, Apgar scores (range, 0-10), birth weight, and small for gestational age (birth weight <10th percentile at the same gestational age); and (6) neonatal disorders: respiratory failure, anemia, septicemia, hypoglycemia or hyperglycemia, respiratory distress syndrome (RDS), pneumonia, and hyperbilirubinemia. Continuous independent variables, including age at pregnancy, prepregnancy BMI, Apgar score, and neonatal birth weight, were converted into categorical variables to decrease the effect of extreme values.

### Predictor Selection

To enhance the computational efficiency of the ANN model, logistic regression analysis was first performed to select important factors. We subsequently incorporated the putative predictive factors, those with *P* < .10, into a multivariable model using the forced entry method. The association of each factor with the risk of MBD was estimated with odds ratios (ORs) and 95% CIs. Variables with statistical significance (*P* < .05) were reserved to build the ANN model.

### Statistical Analysis

#### Dealing With Missing Data

Categorical values were presented as numbers (percentages) and were used to compare MBD and control groups using the χ*^2^* test or Fisher exact test. To investigate the potential attrition bias from the missing data, we repeated this testing for all factors in the full singleton cohort with imputed data before proceeding with the main analyses. The missing data were estimated by multiple imputation by chained equations.

#### ANN Model Construction

A feed-forward ANN framework was applied to construct predictive models. The model structure comprised 3 layers. The input layer had the factors we chose for model construction as neurons, and the output layer had 2 neurons (ie, MBD and non-MBD events). The hidden layer comprises weighted inputs as neurons and produces a classification of the predicted event in the output layer. The total sample was randomized into a training set (70%) for model development and a test set (30%) for validation. The number of hidden layer neurons was determined by 100 iterations, and the BFGS (Broyden, Fletcher, Goldfarb and Shanno) method was applied to determine optimal model parameters.

#### Predictive Model Evaluation

Under the ANN framework, model 1 was first built with significant factors selected from logistic regression models (significant prenatal and postnatal factors). We also constructed a set of ANN models with demographic characteristics and significant variables from different periods: (1) maternal nutritional conditions in model 2, (2) gestational complications and comorbidities and medication use in model 3; (3) all prenatal factors (in items 1 and 2) in model 4; and (4) postnatal factors (birth outcomes and neonatal disorders) in model 5 (eTables 2 and 3 in Supplement 1).

Model performance was reported by approaches of discrimination, calibration, and reclassification. The area under the receiver operating characteristic curve (AUC), accuracy, sensitivity, specificity, positive predictive value, and negative predictive value were calculated. The optimal threshold value for the AUC to distinguish a predictive event was determined by the maximum Youden Index. The model with the highest AUC was considered to have the best discriminative ability. Net reclassification improvement (>0) was used to quantify the risk classification of 2 models with similar AUCs. Calibration plots were constructed to display the calibration performance of each model. We ranked the predictors included by feature importance based on the Gevrey method.

All analyses were performed using R software, version 4.2.2 (R Foundation for Statistical Computing). A 2-sided *P* < .05 was considered statistically significant in the analyses.

## Results

### Participant Characteristics

Of the 10 801 Chinese women who participated in this study (mean [SD] age, 29.7 [3.9] years), 7104 (65.8%) were local residents, 10 600 (98.1%) were of Han ethnicity, and 1001 (9.3%) had uterine scarring (Table 1). A total of 5950 infants were male (55.1%) and 4851 (44.9%) were female. There were 138 neonates (1.3%) with MBD; among them, only 6 (4.3%) were term infants, whereas 8094 control neonates (75.9%) were term infants. Other factors during antenatal and postpartum periods are outlined in eTable 4 in Supplement 1. Distribution patterns of factors in the full singleton cohort with imputed data remained essentially unchanged (eTable 5 in Supplement 1).

**Table 1.** Demographic Characteristics of the MBD and Control Groups

Characteristic	No. (%) of participants		*P* value^a^
	MBD group (n = 138)	Control group (n = 10 663)	
Age at pregnancy, y			
≤20	2 (1.5)	48 (0.5)	.03
21-30	76 (55.0)	6814 (63.9)	
31-40	58 (42.0)	3722 (34.9)	
>40	2 (1.5)	79 (0.7)	
Prepregnancy BMI			
<18.5	10 (7.2)	1358 (12.7)	.001
18.5-23.9	88 (63.8)	7364 (69.1)	
24-27.9	25 (18.1)	1419 (13.3)	
≥28	15 (10.9)	522 (4.9)	
Ethnicity			
Han	136 (98.6)	10 464 (98.1)	.97
Ethnic minority group^b^	2 (1.4)	199 (1.9)	
Occupation			
Employed	108 (78.3)	9255 (86.8)	.003
Unemployed	30 (21.7)	1408 (13.2)	
Region			
Local residents	96 (69.6)	7008 (65.7)	.35
Others	42 (30.4)	3655 (34.3)	
Parity			
Primipara	101 (73.2)	8709 (81.7)	.01
Multipara	37 (26.8)	1954 (18.3)	

Abbreviations: BMI, body mass index (calculated as weight in kilograms divided by height in meters squared); MBD, metabolic bone disease.

^a^ The difference in distribution of each variable between the MBD and control groups was tested with the χ^2^ test or Fisher exact test.

^b^ Ethnic minority group refers to 55 other ethnic groups in China except for the Han ethnicity.

### Putative Predictive Factors

After maternal demographic characteristics were adjusted for, the risk of having MBD offspring was 2.31 (95% CI, 1.19-4.48; *P* = .01) times higher in women who had inadequate folic acid during pregnancy, 3.260 (95% CI, 1.80-5.92; *P* < .001) times higher in those with calcium supplementation, and 0.38 (95% CI, 0.22-0.64; *P* < .001) times lower if taking iron supplements. Magnesium sulfate use in pregnancy (OR, 1.80; 95% CI, 1.05-3.06; *P* = .03) and infants with low birth weight (OR, 5.49; 95% CI, 1.64-18.40; *P* = .006), anemia (OR, 3.04; 95% CI, 1.86-5.14; *P* < .001), septicemia (OR, 3.00; 95% CI, 1.51-5.96; *P* = .002), or RDS (OR, 6.06; 95% CI, 3.17-11.59; *P* < .001) were risk factors for developing MBD (Table 2).

**Table 2.** Putative Predictive Factors for Metabolic Bone Disease Risk From Antenatal to Postpartum Periods

Characteristic	No. (%)	Crude analysis^a^		Adjusted analysis^b^	
		*P* value	OR (95% CI)	*P* value	OR (95% CI)
Demographic characteristics					
Age at pregnancy, y^c^					
21-30	6890 (63.8)	.07	0.27 (0.06-1.12)	.004	0.08 (0.01-0.43)
31-40	3780 (35.0)	.18	0.37 (0.09-1.58)	<.001	0.04 (0.01-0.21)
>40	81 (7.5)	.62	0.61 (0.08-4.46)	.04	0.08 (0.01-0.84)
Prepregnancy BMI^d^					
18.5-23.9	7452 (69.0)	.15	1.62 (0.84-3.13)	.17	1.18 (0.79-3.96)
24-27.9	1444 (13.4)	.02	2.39 (1.15-5.00)	.22	1.78 (0.71-4.47)
≥28	537 (5.0)	.001	3.90 (1.74-8.74)	.46	1.47 (0.53-4.06)
Unemployed^e^	1438 (13.3)	.004	1.83 (1.21-2.75)	.39	1.27 (0.74-2.17)
Multipara >1	1991 (18.4)	.01	1.63 (1.12-2.39)	.09	1.56 (0.94-2.60)
Maternal nutritional conditions					
Deficiency of folic acid	765 (7.1)	<.001	2.53 (1.60-4.02)	.01	2.31 (1.19-4.48)
Deficiency of vitamin D	2575 (23.8)	.03	1.50 (1.05-2.16)	.21	1.36 (0.84-2.19)
Supplementation of iron	3949 (36.6)	.001	0.50 (0.33-0.75)	<.001	0.38 (0.22-0.64)
Supplementation of calcium	1013 (9.4)	.004	1.95 (1.24-3.07)	<.001	3.26 (1.80-5.92)
Gestational complications and comorbidities					
Placenta previa	249 (2.3)	.008	2.66 (1.29-5.50)	.25	1.67 (0.70-3.95)
Placental abruption	165 (1.5)	.001	3.55 (1.63-7.72)	.22	0.51 (0.18-1.48)
Gestational hypertension	999 (9.2)	<.001	2.43 (1.58-3.71)	.40	1.28 (0.72-2.26)
Fever	1577 (14.6)	.03	0.50 (0.27-0.93)	.98	0.99 (0.45-2.18)
Gestational medication use					
Use of dexamethasone	1159 (10.7)	<.001	6.90 (4.90-9.71)	.55	0.86 (0.53-1.41)
Use of magnesium sulfate	1374 (12.7)	<.001	18.02 (12.42-26.16)	.03	1.795 (1.054-3.057)
Use of furosemide	357 (3.3)	<.001	3.40 (1.94-5.97)	.58	0.81 (0.38-1.73)
Birth outcomes					
Prematurity	2701 (25.0)	<.001	69.31 (30.55-157.27)	.21	2.46 (0.60-10.07)
Neonatal Apgar scores ≥7	10 433 (96.6)	<.001	0.24 (0.14-0.41)	.35	0.70 (0.33-1.47)
Neonatal birth weight^f^		<.001		<.001	
Low (1500-2500 g)	1480 (13.7)	<.001	30.90 (15.05-63.42)	.006	5.49 (1.64-18.40)
Very low (1000-1500 g)	208 (1.9)	<.001	545.25 (267.27-1112.33)	<.001	26.33 (7.50-92.42)
Extremely low (<1000 g)	29 (0.3)	<.001	711.77 (265.38-1908.99)	<.001	35.95 (7.78-166.15)
Neonatal disorders					
Neonatal anemia	321 (3.0)	<.001	47.17 (33.00-67.41)	<.001	3.09 (1.86-5.14)
Neonatal septicemia	123 (1.1)	<.001	28.32 (17.85-44.92)	.002	3.00 (1.51-5.96)
Hypoglycemia or hyperglycemia	459 (4.2)	.003	2.38 (1.34-4.25)	.47	0.75 (0.35-1.63)
Neonatal RDS	728 (6.7)	<.001	91.66 (57.19-146.89)	<.001	6.06 (3.17-11.59)
Neonatal hyperbilirubinemia	5634 (52.2)	<.001	0.44 (0.31-0.63)	.20	1.36 (0.85-2.17)

Abbreviations: BMI, body mass index (calculated as weight in kilograms divided by height in meters squared; OR, odds ratio; RDS, respiratory distress syndrome.

^a^ Performed with a univariable logistic regression model.

^b^ Performed with a multivariable logistic regression model.

^c^ Reference to age of 20 years or younger.

^d^ Reference to BMI less than 18.5.

^e^ Reference to employed individuals.

^f^ Reference to non–low birth weight (≥2500 g).

### ANN Predictive Models

**Figure 1.**
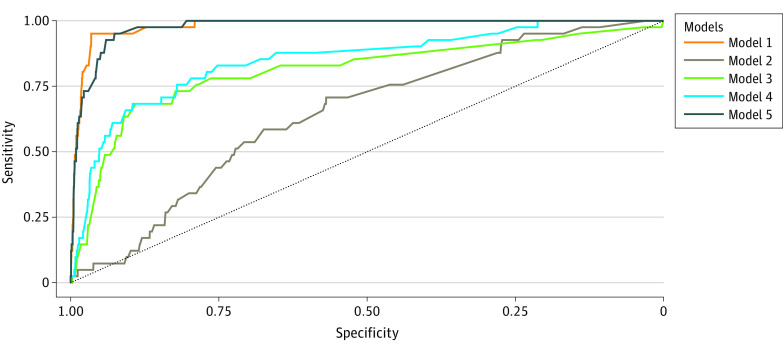
The Receiver Operating Characteristic Curves of 5 Artificial Neural Network Models

A total of 9 significant variables were included in model 1 (significant prenatal and postnatal factors). Model 1 exhibited the highest AUC of 0.981 (95% CI, 0.970-0.992), followed by model 5 (postnatal factors; AUC, 0.977; 95% CI, 0.966-0.988), model 4 (all prenatal factors; AUC, 0.850; 95% CI, 0.785-0.915), model 3 (gestational complications and comorbidities and medication use; AUC, 0.808; 95% CI, 0.726-0.891), and model 2 (maternal nutritional conditions; AUC, 0.647; 95% CI, 0.571-0.723) (Table 3 and Figure 1). The net reclassification improvement was 0.205 (95% CI, 0.067-0.335) when comparing model 1 with model 5, illustrating a better discriminative ability of model 1. Model 1 had a comparable sensitivity to model 5 (both 0.951; 95% CI, 0.885-1.000) but a higher PPV (0.260; 95% CI, 0.190-0.330) than model 5 (0.140; 95% CI, 0.100-0.182), supporting that it could precisely identify neonates at higher risk for MBD with less misclassification of low-risk individuals. Compared with model 3, model 4 included more prenatal factors and had higher sensitivity (model 4: 0.829; 95% CI, 0.714-0.994 vs model 3: 0.683; 95% CI, 0.540-0.825), indicating a possibly stronger ability to recognize infants at risk for MBD before delivery. In addition, model 1 and model 5 were well calibrated according to the calibration plots (eFigures 2-6 in Supplement 1).

**Table 3.** Comparison of the Performance of 5 Artificial Neural Network Models

Model	AUC (95% CI)	Accuracy (95% CI)	Sensitivity (95% CI)	Specificity (95% CI)	PPV (95% CI)	NPV (95% CI)
Model 1^a^	0.981 (0.970-0.992)	0.965 (0.965-0.965)	0.951 (0.885-1.000)	0.965 (0.959-0.972)	0.260 (0.190-0.330)	0.999 (0.998-1.000)
Model 2^b^	0.647 (0.571-0.723)	0.571 (0.571-0.571)	0.707 (0.568-0.847)	0.569 (0.552-0.586)	0.021 (0.013-0.028)	0.993 (0.990-0.997)
Model 3^c^	0.808 (0.726-0.891)	0.888 (0.888-0.888)	0.683 (0.540-0.825)	0.891 (0.880-0.901)	0.074 (0.048-0.100)	0.995 (0.993-0.998)
Model 4^d^	0.850 (0.785-0.915)	0.753 (0.753-0.754)	0.829 (0.714-0.944)	0.752 (0.738-0.767)	0.041 (0.028-0.055)	0.997 (0.995-0.999)
Model 5^e^	0.977 (0.966-0.988)	0.926 (0.926-0.926)	0.951 (0.885-1.000)	0.926 (0.917-0.935)	0.141 (0.100-0.182)	0.999 (0.998-1.000)

Abbreviations: AUC, area under the curve; NPV, negative predictive value; PPV, positive predictive value.

^a^ Model 1 included significant prenatal and postnatal factors (ie, age at pregnancy, deficiency of folic acid, supplementation of iron, supplementation of calcium, use of magnesium sulfate, neonatal birth weight, neonatal anemia, neonatal septicemia, and neonatal respiratory distress syndrome).

^b^ Model 2 included maternal nutritional condition factors (ie, age at pregnancy, prepregnancy body mass index, occupation, parity, deficiency of folic acid, deficiency of vitamin D, supplementation of iron, and supplementation of calcium).

^c^ Model 3 included gestational complications and comorbidities and medication use factors (ie, age at pregnancy, prepregnancy body mass index, occupation, parity, gestational hypertension, use of dexamethasone, and use of magnesium sulfate).

^d^ Model 4 included all prenatal factors (ie, age of pregnancy, prepregnancy body mass index, occupation, parity, deficiency of folic acid, supplementation of iron, supplementation of calcium, gestational hypertension, use of dexamethasone, and use of magnesium sulfate).

^e^ Model 5 included postnatal factors (ie, age at pregnancy, prepregnancy body mass index, occupation, parity, neonatal birth weight, neonatal anemia, neonatal septicemia, and neonatal respiratory distress syndrome).

**Figure 2.**
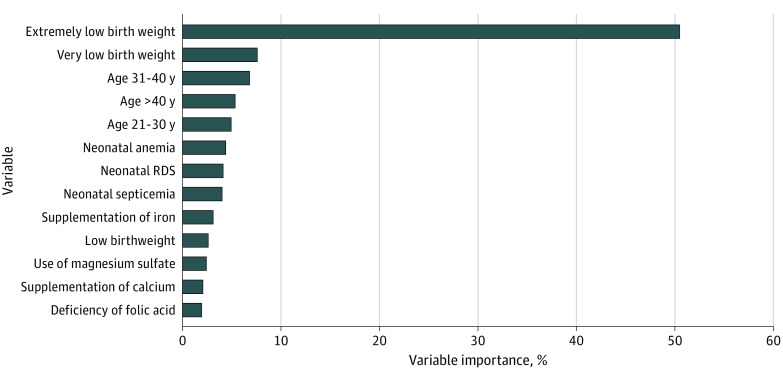
Variable Importance for Model 1 for Predicting the Risk of Metabolic Bone Disease in Neonates Model 1 includes significant prenatal and postnatal factors (ie, age at pregnancy, deficiency of folic acid, supplementation of iron, supplementation of calcium, use of magnesium sulfate, neonatal birth weight, neonatal anemia, neonatal septicemia, and neonatal respiratory distress syndrome [RDS]).

A list of factors included in each model and their importance is presented in eTable 6 in Supplement 1. The variable importance plot of model 1 (Figure 2) suggested that birth weight, maternal age at pregnancy, and neonatal disorders are the most important characteristics for predicting an infant at risk for MBD; among these factors, ELBW (importance, 50.5%) followed by VLBW (importance, 7.6%) were the most powerful predictive characteristics. The factors that ranked first were ELBW (importance, 15.1%) in model 1 and use of magnesium sulfate (importance, 21.2%) in model 4 (eTable 6 and eFigures 7-10 in Supplement 1).

## Discussion

In this diagnostic study, 5 ANN models were proposed using different exposed factors obtained from prenatal and/or postnatal periods to make early predictions regarding neonates at risk for MBD. Model 1 (significant prenatal and postnatal factors) and model 5 (postnatal factors) showed the best performance. If prenatal factors are unavailable, we recommend that model 1 and model 5 be used in clinical practice (postdelivery prediction) and model 4 (all prenatal factors) be used before delivery in the study population.

The 3 suggested predictive models had an excellent discriminative ability, with a superior AUC of 0.981 in model 1 and 0.977 in model 5. Currently, to our knowledge, no research has focused on making predictions for neonates at risk for MBD using machine learning techniques; therefore, we could not compare model performance. However, many researchers focused their attention on developing practical screening tools for bone diseases in adults, such as osteoporosis, using ANN methods and acknowledged its predictive power, especially in mass screening application.^25,26^ Another study^27^ found that ANN outperformed linear regression models in prediction of bone density among postmenopausal women when more variables were imported. However, it is challenging for ANN to scale up to recordings of thousands of neurons and to provide medical explanations pertaining to each independent variable as linear or logistic models do.^28,29^ In addition, ANN needs a long processing time for large neural networks. This study considered many factors (neurons), and the sample size had rigorous requirements when putting all these factors into ANN models. Therefore, only the variables selected by logistic regression analysis were used as input variables.

Our findings revealed that ELBW and VLBW had a significant influence on the risk of MBD during infancy. Previous studies^7,19,30,31^ identified birth weight along with gestational age as the strongest risk factors for MBD in preterm infants and recommended that infants with birth weights less than 1500 g be screened for MBD.^32^ Approximately 80% of fetal bone mineral accretion occurs during the last 3 months of pregnancy, and preterm neonates will miss, partially or fully, this critical period of bone growth.^33^ Prematurity, however, was not identified as a significant predictor in this study. We observed that 95.7% of neonates with MBD were preterm; therefore, birth weight might be more sensible than prematurity for prediction. Although the gestational age was not included in the analysis, its role might have been implied by prematurity combined with birth weight. We also found no association of uterine scarring, a potential risk factor for preterm delivery,^34^ with the risk of MBD.

Intrauterine mineral deficit can worsen in the postnatal period due to neonatal disorders responsible for associated bone loss.^2,35^ Consistent with previous results,^36^ we found that neonatal RDS, anemia, and septicemia were associated with increased risk of MBD, which could be a result of restriction in bone growth due to disease relevant therapies and induced stress responses.^37,38^ Among antenatal risk factors, the use of magnesium sulfate was the most significant factor associated with the risk of MBD in infants (model 4), highlighting its detrimental influence on neonatal bone health.^39,40^ Maternal magnesium can influence fetal parathyroid hormone balance and compete with calcium metabolism via crossing the placenta to fetus, consequently causing bone atrophy in neonates.^40^ The deficiency of folic acid may influence normal bone formation and growth,^41^ and maternal iron and calcium supplementation could help rule out or identify MBD development in neonates. Of note, we detected a calcium-associated risk for MBD through multivariable analysis. This risk may indicate largely inadequate calcium storage during gestation.

### Strengths and Limitations

Our study had several strengths. To our knowledge, this was the first study to use artificial intelligence models for MBD risk prediction in very early life. Given the data availability in clinical settings, we developed a set of predictive tools tailored to different scenarios from antenatal to postnatal periods and selected the optimal ones for application. When available, more than 95% of MBD could be accurately predicted using fewer than 10 variables. The predictive model we developed is a rapid and effective tool that could be applied to daily clinical practice, such as by developing a computerized system for risk screening purposes. Another advantage was that we used data from a long-established cohort of pregnant women (2012-2021) with a large sample size (n = 10 801). The prospective design ensured the reliability of exposed factors obtained and their associations with MBD risk. On the basis of our results, we were able to identify some clinically relevant predictive factors, such as birth weight and maternal use of magnesium sulfate. Identification of these factors could allow for special monitoring of those with high-risk factors of MBD, such as infants with low birth weight and pregnant women who are at risk of delivering preterm or growth-restricted infants. Furthermore, MBD is not a condition limited to the preterm infant. Unlike most MBD reports, which focus on premature births,^5,17,18^ our results could be generalized to both term and preterm infants.

Some limitations should also be noted. First, because of the absence of a bone imaging method, the diagnosis of MBD was made by biochemical criteria based on serum alkaline phosphatase levels. However, radiographs only reveal bone mineralization when its reduction is up to 20% to 40%^42^ and are of limited use to in the early diagnosis of MBD. Second, postnatal mineral supply,^30^ type of feeding,^43^ parenteral nutrition,^12^ and some important nutrient intake during pregnancy (eg, phosphorus, magnesium, zinc, potassium, and protein) may also be involved in bone metabolism. These indicators require further research and generative evidence before claims are made. Given the study purpose and strong performance of our predictive models, we suggest that current data are sufficient to meet the goal. Third, this study included only women with singleton pregnancy in consideration of the complicated mechanism of multiple-birth pregnancy that may introduce unknown confounders and lead to unexplained results. Specialized, well-designed observational studies are needed to investigate the association of multiple births with the risk of MBD. Fourth, higher risk of mortality in preterm (compared with term) infants might bias the results. However, because of a high level of medical care, such survivorship bias might not exist in this study because the survival rate of neonates approaches 100% within 72 hours after birth at our institute, at which time infants are screened for MBD. Of note, these factors are applicable to the population of study but need to be tested in other populations and countries.

## Conclusions

In this diagnostic study of 10 801 participants, ANN appeared to be a simple and efficient tool to identify neonates at risk of MBD and could therefore effectively help with preventive efforts. Combining prenatal and postnatal factors or using postnatal exposures alone provided the most precise prediction. The most important predictor was ELBW. The use of magnesium sulfate during pregnancy was a significant predictor for the risk of MBD when postnatal factors were unavailable. However, further investigations are warranted to validate these findings in other populations.

## Notes

Supplement 1. **eMethods.** Estimation of Sample Size **eTable 1.** Factors Collected in the Study and the Missing Numbers **eTable 2.** The Associations of Prenatal Predictive Factors With the Risk of MBD in Neonates **eTable 3.** The Associations of Postnatal Predictive Factors With the Risk of MBD in Neonates **eTable 4.** Clinical Characteristics of MBD Group and Control Group During Antenatal and Postpartum Periods **eTable 5.** Distribution of Factors in MBD Group and Control Group: Analysis for Missing Data **eTable 6.** Factors Included in Five ANN Models and Their Proportion of Variable Importance **eFigure 1.** Flow Diagram of the Study Population **eFigure 2.** The Calibration Plot of Model 1 **eFigure 3.** The Calibration Plot of Model 2 **eFigure 4.** The Calibration Plot of Model 3 **eFigure 5.** The Calibration Plot of Model 4 **eFigure 6.** The Calibration Plot of Model 5 **eFigure 7.** The Variable Importance of Model 2 for Predicting the Risk of MBD in Neonates **eFigure 8.** The Variable Importance of Model 3 for Predicting the Risk of MBD in Neonates **eFigure 9.** The Variable Importance of Model 4 for Predicting the Risk of MBD in Neonates **eFigure 10.** The Variable Importance of Model 5 for Predicting the Risk of MBD in Neonates
eReference

Supplement 2.
Data Sharing Statement
