# Supplementary material for: Artificial Neural Network Modeling to Predict Neonatal Metabolic Bone Disease in the Prenatal and Postnatal Periods
Source: JAMA Netw Open. 2023 Jan 23;6(1):e2251849. doi: 10.1001/jamanetworkopen.2022.51849 (PMC9871802; doi:10.1001/jamanetworkopen.2022.51849)
Supplement: Supplement 1. — eMethods. Estimation of Sample Size eTable 1. Factors Collected in the Study and the Missing Numbers eTable 2. The Associations of Prenatal Predictive Factors With the Risk of MBD in Neonates eTable 3. The Associations of Postnatal Predictive Factors With the Risk of MBD in Neonates eTable 4. Clinical Characteristics of MBD Group and Control Group During Antenatal and Postpartum Periods eTable 5. Distribution of Factors in MBD Group and Control Group: Analysis for Missing Data eTable 6. Factors Included in Five ANN Models and Their Proportion of Variable Importance eFigure 1. Flow Diagram of the Study Population eFigure 2. The Calibration Plot of Model 1 eFigure 3. The Calibration Plot of Model 2 eFigure 4. The Calibration Plot of Model 3 eFigure 5. The Calibration Plot of Model 4 eFigure 6. The Calibration Plot of Model 5 eFigure 7. The Variable Importance of Model 2 for Predicting the Risk of MBD in Neonates eFigure 8. The Variable Importance of Model 3 for Predicting the Risk of MBD in Neonates eFigure 9. The Variable Importance of Model 4 for Predicting the Risk of MBD in Neonates eFigure 10. The Variable Importance of Model 5 for Predicting the Risk of MBD in Neonates eReference [file jamanetwopen-e2251849-s001.pdf]

Supplementary Online Content

Jiang H, Guo J, Li J, et al. Artificial neural network modeling to predict neonatal metabolic bone disease in the prenatal and postnatal periods. *JAMA Netw Open*. 2023;6(1):e2251849. doi:10.1001/jamanetworkopen.2022.51849

eMethods. Estimation of Sample Size

eTable 1. Factors Collected in the Study and the Missing Numbers

eTable 2. The Associations of Prenatal Predictive Factors With the Risk of MBD in Neonates

eTable 3. The Associations of Postnatal Predictive Factors With the Risk of MBD in Neonates

eTable 4. Clinical Characteristics of MBD Group and Control Group During Antenatal and Postpartum Periods

eTable 5. Distribution of Factors in MBD Group and Control Group: Analysis for Missing Data

eTable 6. Factors Included in Five ANN Models and Their Proportion of Variable Importance

eFigure 1. Flow Diagram of the Study Population

eFigure 2. The Calibration Plot of Model 1

eFigure 3. The Calibration Plot of Model 2

eFigure 4. The Calibration Plot of Model 3

eFigure 5. The Calibration Plot of Model 4

eFigure 6. The Calibration Plot of Model 5

eFigure 7. The Variable Importance of Model 2 for Predicting the Risk of MBD in Neonates

eFigure 8. The Variable Importance of Model 3 for Predicting the Risk of MBD in Neonates

eFigure 9. The Variable Importance of Model 4 for Predicting the Risk of MBD in Neonates

eFigure 10. The Variable Importance of Model 5 for Predicting the Risk of MBD in Neonates

eReference

This supplementary material has been provided by the authors to give readers additional information about their work.

© 2023 Jiang H et al. *JAMA Network Open.*

eMethods: estimation of sample size

The minimum sample size required for this study was estimated using the method proposed by Riley et al[1] in four steps. An estimate was generated in each step, and the maximum estimate among them was chosen as the final sample size. Four steps and the corresponding formulas are shown below:

Step 1: $n = \left(\frac{Z_{1-\alpha/2}}{\delta}\right) \times p \times (1-p)$ ①

Step 2: $n = exp\left(\frac{-0.508+0.259ln\phi+0.504lnP-lnMAPE}{0.544}\right)$ ②

Step 3: $n = \frac{P}{(S-1)ln\left(1-\frac{R_{CS}^2}{S}\right)}$ ③

where p is the prevalence of metabolic bone disease (MBD) in neonates, δ is the margin of error, α is the Type I error, ϕ is the anticipated MBD proportion, P is the number of candidate predictors, *MAPE* is the average error in the model's estimated outcome, S is the expected uniform shrinkage factor, and R_{CS}^2 reflects the noise ratio of model.

In Step 4, S was first calculated based on the following formula④ and introduced into formula③ to estimate the sample size:

$S = \frac{R_{CS}^2}{R_{CS}^2+\delta max(R_{CS}^2)}$ ④

We used the "pmsampsize" Package in R 4.2.2 (R Foundation, Vienna, Austria) to perform the procedure of estimation for sample size. According to literatures and our observation from clinical practice, we assumed that the p in this study was 0.05. The minimum sample sizes required for logistic regression model and for artificial neural network (ANN) predictive model were estimated, respectively.

(1) Sample size for logistic regression model:
Assumed that R_{CS}^2 was 0.2. When incorporating all of the interested factors (i.e., P was 38) as variables into logistic regression models for predictor selection, the minimum sample size required for this study was 2059, including 103 MBD cases.

(2) Sample size for ANN predictive model:
According to the results of logistic regression models, the maximum number of predictors selected for the construction of ANN models was 10, and the R_{CS}^2 was 0.068. The minimum sample size required for a prediction by ANN was 1273, including 64 MBD cases.

The number of participants included in this study has met the above criteria.

© 2023 Jiang H et al. *JAMA Network Open.*

Supplemental Tables

eTable 1. Factors collected in the study and the missing numbers

Collected factors	Groups and assignment	Missing number
**Demographics**		
Age at pregnancy (y)	1=age ≤20; 2=age 21-30; 3=age 31-40; 4=age >40	0
Pre-pregnancy BMI (kg/m^2)	1= BMI <18.5; 2=BMI 18.5-23.9; 3=BMI 24-27.9; 4= BMI ≥28	3062
Ethnicity (in resident's ID card)	1=Han; 2=Ethnic minorities	37
Occupation	1=Employed; 2=Unemployed	137
Region	1=Local residents; 2= Others	58
Parity	1=Primipara; 2=Multipara	37
Uterine scarring	1=Yes; 0=No	37
**Nutritional conditions during pregnancy**		
Deficiency of folic acid	1=Yes (<10 nmol/L); 0=No	3050
Deficiency of ferritin	1=Yes (<15 μg/mL); 0=No	3053
Deficiency of vitamin D	1=Yes (<30 ng/mL); 0=No	3054
Gestational anemia	1=Yes (serum hemoglobin <110 g/L); 0=No	3038
Supplementation of folic acid	1=Yes; 0=No	0
Supplementation of iron	1=Yes; 0=No	0
Supplementation of calcium	1=Yes; 0=No	0
Supplementation of vitamin D	1=Yes; 0=No	0
**Gestational complications/comorbidities**		
Placenta previa	1=Yes; 0=No	37
Placental abruption	1=Yes; 0=No	37
Gestational diabetes	1=Yes; 0=No	37
Gestational hypertension	1=Yes; 0=No	37
Renal disease	1=Yes; 0=No	37
Fever	1=Yes; 0=No	37
**Gestational medication use**		
Use of dexamethasone	1=Yes; 0=No	0
Use of magnesium sulfate	1=Yes; 0=No	0
Use of antibiotics	1=Yes; 0=No	0
Use of furosemide	1=Yes; 0=No	0
**Birth outcomes**		
Prematurity	1=Yes (gestational age <37 weeks); 0=No	0
Neonatal sex	1=Male; 2=Female	10
Neonatal Apgar scores	1=Low (<7); 0= Normal (≥7)	39

eTable 1. Factors collected in the study and the missing numbers (continued)

Collected factors	Groups and assignment	Missing number
**Birth outcomes**		
Neonatal birthweight	1=Not low (≥2500 g); 2=Low (1500-2500 g); 3=Very low (1000-1500 g); 4=Extremely low (<1000 g)	5
SGA	1=Yes; 0=No	0
**Neonatal disorders**		
Neonatal respiratory failure	1=Yes; 0=No	0
Neonatal anemia	1=Yes; 0=No	0
Neonatal septicemia	1=Yes; 0=No	0
Hypoglycemia/hyperglycemia	1=Yes; 0=No	0
Neonatal RDS	1=Yes; 0=No	0
Neonatal pneumonia	1=Yes; 0=No	0
Neonatal hyperbilirubinemia	1=Yes; 0=No	0

Abbreviations: MBD, metabolic bone disease; BMI, body mass index; SGA, small for gestational age; RDS, respiratory distress syndrome.

Ethnic minorities refer to 55 other ethnic groups in China except for the Han ethnicity.

© 2023 Jiang H et al. *JAMA Network Open.*

eTable 2. The associations of prenatal predictive factors with the risk of MBD in neonates

Factors	cOR[a] (95% CI)	aOR[b] (95% CI)	aOR[c] (95% CI)
**Nutritional conditions during pregnancy**			
Deficiency of folic acid	2.532 (1.596-4.018)	2.133 (1.318-3.451)	2.126 (1.267-3.565)
Deficiency of ferritin	1.396 (0.837-2.329)		
Deficiency of vitamin D	1.504 (1.048-2.157)	1.539 (1.063-2.229)	1.421 (0.962-2.097)
Gestational anemia	1.097 (0.649-1.853)		
Supplementation of folic acid	1.370 (0.736-2.550)		
Supplementation of iron	0.499 (0.334-0.745)	0.454 (0.302-0.683)	0.355 (0.231-0.547)
Supplementation of calcium	1.954 (1.243-3.072)	2.081 (1.315-3.294)	1.839 (1.132-2.988)
Supplementation of vitamin D	1.160 (0.799-1.685)		
**Gestational complications/comorbidities/medication use**			
Placenta previa	2.661 (1.289-5.496)	0.906 (0.421-1.949)	1.166 (0.536-2.539)
Placental abruption	3.553 (1.634-7.722)	2.120 (0.930-4.831)	2.086 (0.898-4.846)
Gestational diabetes	0.913 (0.548-1.522)		
Gestational hypertension	2.425 (1.584-3.712)	0.446 (0.274-0.725)	0.439 (0.268-0.717)
Renal disease	3.719 (0.891-15.518)		
Fever	0.503 (0.271-0.934)	0.782 (0.412-1.484)	0.786 (0.410-1.505)
Use of dexamethasone	6.901 (4.904-9.712)	1.543 (1.028-2.315)	1.667 (1.106-2.513)
Use of magnesium sulfate	18.024 (12.420-26.155)	17.779 (11.273-28.042)	17.645 (11.158-27.902)
Use of antibiotics	0.815 (0.579-1.148)		
Use of furosemide	3.397 (1.935-5.965)	1.169 (0.638-2.140)	1.207 (0.651-2.237)

Abbreviations: MBD, metabolic bone disease; OR, odd ratio; cOR, crude OR; aOR, adjusted OR; CI, confidence interval.

[a] Unadjusted OR estimated using univariable logistic regression model.

[b] Adjusted only for maternal demographics using multivariable logistic regression model. The significant variables are chosen to build Model 2/Model 3.

[c] Adjusted for maternal demographics and other prenatal influence factors during pregnancy using multivariate logistic regression model. The significant variables are chosen to build Model 4 as prenatal predictors.

© 2023 Jiang H et al. *JAMA Network Open.*

eTable 3. The associations of postnatal predictive factors with the risk of MBD in neonates

Factors	cOR[a] (95% CI)	aOR[b] (95% CI)
Prematurity	69.314 (30.548-157.274)	2.891 (0.727-11.493)
Neonatal sex (male)	1.194 (0.854-1.671)	
Neonatal Apgar scores (<7)	0.242 (0.144-0.407)	0.893 (0.441-1.809)
Neonatal birthweight [c]		
Low (1500-2500 g)	30.896 (15.051-63.422)	6.101 (1.893-19.664)
Very low (1000-1500 g)	545.247 (267.272-1112.329)	28.999 (8.547-98.396)
Extremely low (<1000 g)	711.765 (265.381-1908.987)	30.847 (7.102-133.974)
SGA	1.871 (0.867-4.038)	
Neonatal respiratory failure	1.017 (0.140-7.364)	
Neonatal anemia	47.165 (32.998-67.412)	3.005 (1.847-4.889)
Neonatal septicemia	28.316 (17.850-44.919)	2.557 (1.328-4.922)
Hypoglycemia/hyperglycemia	2.382 (1.335-4.251)	0.809 (0.385-1.704)
Neonatal RDS	91.660 (57.194-146.893)	6.027 (3.236-11.225)
Neonatal pneumonia	1.019 (0.698-1.489)	
Neonatal hyperbilirubinemia	0.439 (0.307-0.628)	1.319 (0.846-2.056)

Abbreviations: MBD, metabolic bone disease; OR, odd ratio; cOR, crude OR; aOR, adjusted OR; CI, confidence interval; SGA, small for gestational age; RDS, respiratory distress syndrome.

[a] Unadjusted OR estimated using univariable logistic regression model.

[b] Adjusted for maternal demographics using multivariable logistic regression model. The significant variables are chosen to build Model 5 as postnatal predictors.

[c] Reference to non-low birthweight (≥2500 g).

© 2023 Jiang H et al. *JAMA Network Open.*

eTable 4. Clinical characteristics of MBD group and control group during antenatal and postpartum periods

**Factors**	**MBD (n=138), No. (%)**	**Controls (n=10 663), No. (%)**	***P* value [a]**
**Previous pregnancy history**			
Uterine scarring	15 (10.9)	986 (9.2)	.51
**Nutritional conditions during pregnancy**			
Deficiency of folic acid	22 (15.9)	743 (7.0)	<.001
Deficiency of ferritin	17 (12.3)	975 (9.1)	.20
Deficiency of vitamin D	44 (31.9)	2531 (23.7)	.03
Gestational anemia	16 (11.6)	1139 (10.7)	.73
Supplementation of folic acid	11 (8.0)	634 (6.0)	.32
Supplementation of iron	31 (22.5)	3918 (36.7)	.001
Supplementation of calcium	23 (16.7)	990 (9.3)	.003
Supplementation of vitamin D	39 (28.3)	2703 (25.4)	.44
**Gestational complications/comorbidities**			
Placenta previa	8 (5.8)	241 (2.3)	.01
Placental abruption	7 (5.1)	158 (1.5)	.002
Gestational diabetes	17 (12.3)	1422 (13.3)	.73
Gestational hypertension	27 (19.6)	972 (9.1)	<.001
Renal disease	2 (1.5)	42 (0.4)	.21
Fever	11 (8.0)	1566 (14.7)	.03
**Gestational medication use**			
Use of dexamethasone	61 (44.2)	1098 (10.3)	<.001
Use of magnesium sulfate	98 (71.0)	1276 (12.0)	<.001
Use of antibiotics	82 (59.4)	6850 (64.2)	.24
Use of furosemide	14 (10.1)	343 (3.2)	<.001
**Birth outcomes**			
Prematurity	132 (95.7)	2569 (24.1)	<.001
Neonatal sex			
Male	70 (50.7)	5880 (55.1)	.30
Female	68 (49.3)	4783 (44.9)	
Neonatal Apgar scores			
Low (<7)	17 (12.3)	351 (3.3)	<.001
Normal (≥7)	121 (87.7)	10312 (96.7)	

© 2023 Jiang H et al. *JAMA Network Open.*

eTable 4. Clinical characteristics of MBD group and control group during antenatal and postpartum periods (continued)

**Factors**	**MBD (n=138), No. (%)**	**Controls (n=10 663), No. (%)**	***P* value [a]**
**Birth outcomes**			
Neonatal birthweight			
Not low (≥2500 g)	9 (6.5)	9075 (85.1)	<.001
Low (1500-2500 g)	44 (31.9)	1436 (13.5)	
Very low (1000-1500 g)	73 (52.9)	135 (1.3)	
Extremely low (<1000 g)	12 (8.7)	17 (0.1)	
SGA	7 (5.1)	296 (2.8)	.17
**Neonatal disorders**			
Neonatal respiratory failure	1 (0.7)	76 (0.7)	>.99
Neonatal anemia	73 (52.9)	248 (2.33)	<.001
Neonatal septicemia	28 (20.3)	95 (0.9)	<.001
Hypoglycemia/hyperglycemia	13 (9.4)	446 (4.2)	.002
Neonatal RDS	117 (84.8)	611 (5.7)	<.001
Neonatal pneumonia	37 (26.8)	2819 (26.4)	.92
Neonatal hyperbilirubinemia	45 (32.6)	5589 (52.4)	<.001

Abbreviations: MBD, metabolic bone disease; SGA, small for gestational age; RDS, respiratory distress syndrome.

[a] The difference in distribution of each variable between MBD and control groups was tested by Chi-square test or Fisher exact test.

© 2023 Jiang H et al. *JAMA Network Open.*

eTable 5. Distribution of factors in MBD group and control group: analysis for missing data

Factors	MBD (n=211), No. (%)	Controls (n=13 748), No. (%)	*P* value [a]
**Demographic characteristics**			
Age at pregnancy (y)			.009
≤20	2 (0.9)	74 (0.5)	
21-30	110 (52.1)	8605 (62.6)	
31-40	95 (45.0)	4954 (36.0)	
>40	4 (1.9)	115 (0.8)	
Pre-pregnancy BMI (kg/m^2)			.001
<18.5	17 (8.1)	1739 (12.6)	
18.5-23.9	134 (63.5)	9454 (68.8)	
24-27.9	40 (19.0)	1896 (13.8)	
≥28	20 (9.5)	659 (4.8)	
Ethnicity			.45
Han	209 (99.1)	13485 (98.1)	
Ethnic minorities [b]	2 (0.9)	263 (1.9)	
Occupation			.05
Employed	179 (84.8)	12257 (89.2)	
Unemployed	32 (15.2)	1491 (10.8)	
Region			.07
Local residents	136 (64.5)	9653 (70.2)	
Others	75 (35.5)	4095 (29.8)	
Parity			<.001
1	150 (71.1)	11233 (81.7)	
>1	61 (28.9)	2515 (18.3)	
Uterine scarring			.37
Yes	24 (11.4)	1314 (9.6)	
No	187 (88.6)	12434 (90.4)	
**Nutritional conditions during pregnancy**			
Deficiency of folic acid	35 (16.6)	961 (7.0)	<.001
Deficiency of ferritin	24 (11.4)	1277 (9.3)	.30
Deficiency of vitamin D	67 (31.8)	3473 (25.3)	.03
Gestational anemia	25 (11.8)	1368 (10.0)	.36
Supplementation of folic acid	14 (6.6)	803 (5.8)	.63
Supplementation of iron	42 (19.9)	5164 (37.6)	<.001
Supplementation of calcium	34 (16.1)	1251 (9.1)	<.001
Supplementation of vitamin D	42 (19.9)	3309 (24.1)	.16
**Gestational complications/comorbidities**			
Placenta previa	12 (5.7)	358 (2.6)	.006

© 2023 Jiang H et al. *JAMA Network Open.*

eTable 5. Distribution of factors in MBD group and control group: analysis for missing data (continued)

Factors	MBD (n=211), No. (%)	Controls (n=13 748), No. (%)	*P* value [a]
**Gestational complications/comorbidities**			
Placental abruption	15 (7.1)	221 (1.6)	<.001
Gestational diabetes	29 (13.7)	1844 (13.4)	.89
Gestational hypertension	39 (18.5)	1249 (9.1)	<.001
Renal disease	2 (0.9)	72 (0.5)	.40
Fever	16 (7.6)	1930 (14.0)	.007
**Gestational medication use**			
Use of dexamethasone	79 (37.4)	1537 (11.2)	<.001
Use of magnesium sulfate	117 (55.5)	1634 (11.9)	<.001
Use of antibiotics	123 (58.3)	8819 (64.1)	.08
Use of furosemide	17 (8.1)	448 (3.3)	<.001
**Birth outcomes**			
Prematurity	204 (96.7)	3512 (25.5)	<.001
Neonatal sex			.35
Male	110 (52.1)	7613 (55.4)	
Female	101 (47.9)	6135 (44.6)	
Neonatal Apgar scores			<.001
Low (<7)	32 (15.2)	464 (3.4)	
Normal (≥7)	179 (84.8)	13284 (96.6)	
Neonatal birthweight			<.001
Not low (≥2500 g)	11 (5.2)	11420 (83.1)	
Low (1500-2500 g)	53 (25.1)	2046 (14.9)	
Very low (1000-1500 g)	109 (51.7)	237 (1.7)	
Extremely low (<1000 g)	38 (18.0)	45 (0.3)	
SGA	10 (4.7)	386 (2.8)	.09
**Neonatal disorders**			
Neonatal respiratory failure	3 (1.4)	175 (1.3)	.85
Neonatal anemia	118 (55.9)	375 (2.7)	<.001
Neonatal septicemia	44 (20.9)	127 (0.9)	<.001
Hypoglycemia/hyperglycemia	19 (9.0)	631 (4.6)	.003
Neonatal RDS	181 (85.8)	946 (6.9)	<.001
Neonatal pneumonia	62 (29.4)	3862 (28.1)	.68
Neonatal hyperbilirubinemia	67 (31.8)	6783 (49.3)	<.001

Abbreviations: MBD, metabolic bone disease; BMI, body mass index; SGA, small for gestational age; RDS, respiratory distress syndrome.

[a] Analysis was performed in the full singleton cohort with multiple imputed data using multiple imputation by chained equations. The difference in distribution of each variable between MBD and control groups was tested by Chi-square test or Fisher exact test.

[b] Ethnic minorities refer to 55 other ethnic groups in China except for the Han ethnicity.

© 2023 Jiang H et al. *JAMA Network Open.*

eTable 6. Factors included in five ANN models and their proportion of variable importance

Factors included	Variable items	Variable importance (%) [a]
**Model 1 (significant pre- and postnatal factors)**		
Age at pregnancy	Age 21-30 y	5.0
	Age 31-40 y	6.8
	Age >40 y	5.4
Deficiency of folic acid	Deficiency of folic acid	2.0
Supplementation of iron	Supplementation of iron	3.1
Supplementation of calcium	Supplementation of calcium	2.1
Use of magnesium sulfate	Use of magnesium sulfate	2.4
Neonatal birthweight	Low (1500-2500 g)	2.6
	Very low (1000-1500 g)	7.6
	Extremely low (<1000 g)	50.5
Neonatal anemia	Neonatal anemia	4.4
Neonatal septicemia	Neonatal septicemia	4.0
Neonatal RDS	Neonatal RDS	4.1
**Model 2 (maternal nutritional condition)**		
Age at pregnancy	Age 21-30 y	10.6
	Age 31-40 y	10.2
	Age >40 y	4.2
Pre-pregnancy BMI	18.5-23.9 km/m^2	4.5
	24-27.9 km/m^2	8.9
	≥28 km/m^2	18.4
Occupation	Unemployed	9.5
Parity	Multipara	7.8
Deficiency of folic acid	Deficiency of folic acid	8.3
Deficiency of vitamin D	Deficiency of vitamin D	3.7
Supplementation of iron	Supplementation of iron	7.8
Supplementation of calcium	Supplementation of calcium	6.1
**Model 3 (gestational complications/comorbidities and medication use)**		
Age at pregnancy	Age 21-30 y	5.9
	Age 31-40 y	9.0
	Age >40 y	10.9
Pre-pregnancy BMI	18.5-23.9 km/m^2	2.8
	24-27.9 km/m^2	6.7
	≥28 km/m^2	13.0
Occupation	Unemployed	6.6

© 2023 Jiang H et al. *JAMA Network Open.*

eTable 6. Factors included in five ANN models and their proportion of variable importance (continued)

Factors included	Variable items	Variable importance (%) [a]
**Model 3 (gestational complications/comorbidities and medication use)**		
Parity	Multipara	7.6
Gestational hypertension	Gestational hypertension	6.5
Use of dexamethasone	Use of dexamethasone	4.7
Use of magnesium sulfate	Use of magnesium sulfate	26.3
**Model 4 (all prenatal factors)**		
Age at pregnancy	Age 21-30 y	6.3
	Age 31-40 y	8.3
	Age >40 y	8.2
Pre-pregnancy BMI	18.5-23.9 km/m^2	3.3
	24-27.9 km/m^2	5.2
	≥28 km/m^2	10.8
Occupation	Unemployed	5.7
Parity	Multipara	5.3
Deficiency of folic acid	Deficiency of folic acid	6.6
Supplementation of iron	Supplementation of iron	6.7
Supplementation of calcium	Supplementation of calcium	2.4
Gestational hypertension	Gestational hypertension	4.9
Use of dexamethasone	Use of dexamethasone	5.0
Use of magnesium sulfate	Use of magnesium sulfate	21.3
**Model 5 (postnatal factors)**		
Age at pregnancy	Age 21-30 y	9.3
	Age 31-40 y	12.2
	Age >40 y	9.1
Pre-pregnancy BMI	18.5-23.9 km/m^2	1.4
	24-27.9 km/m^2	2.7
	≥28 km/m^2	3.4
Occupation	Unemployed	2.8
Parity	Multipara	2.0
Neonatal birthweight	Low (1500-2500 g)	8.1
	Very low (1000-1500 g)	14.5
	Extremely low (<1000 g)	15.1
Neonatal anemia	Neonatal anemia	6.1
Neonatal septicemia	Neonatal septicemia	6.7
Neonatal RDS	Neonatal RDS	6.6

Abbreviations: MBD, metabolic bone disease; RDS, respiratory distress syndrome; BMI, body mass index.

[a] The proportion of variable importance was estimated using Gevrey's method.

© 2023 Jiang H et al. *JAMA Network Open.*

Supplemental Figures

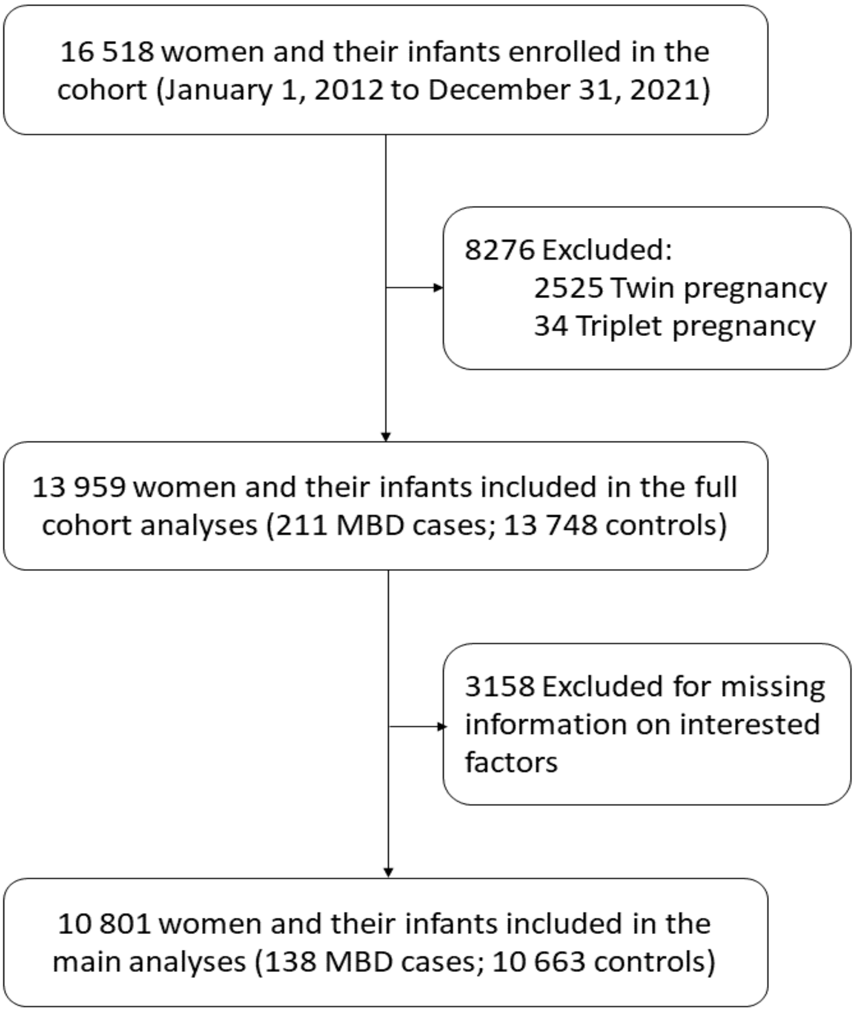

eFigure 1. Flow diagram of the study population

© 2023 Jiang H et al. *JAMA Network Open.*

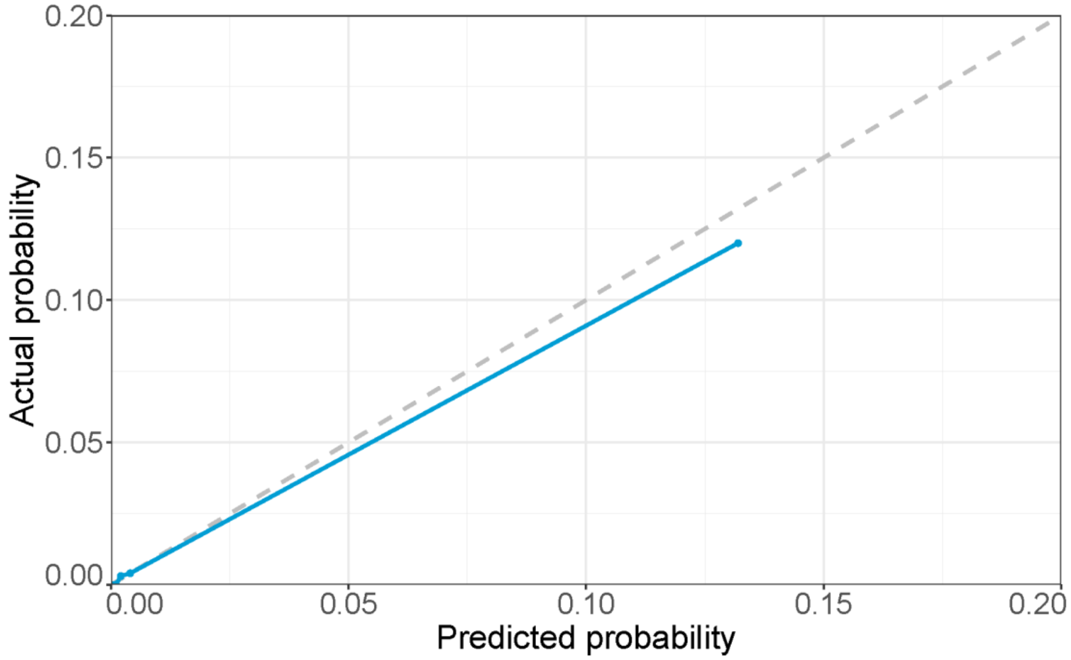

eFigure 2. The calibration plot of Model 1. Model 1, including significant pre- and postnatal factors.

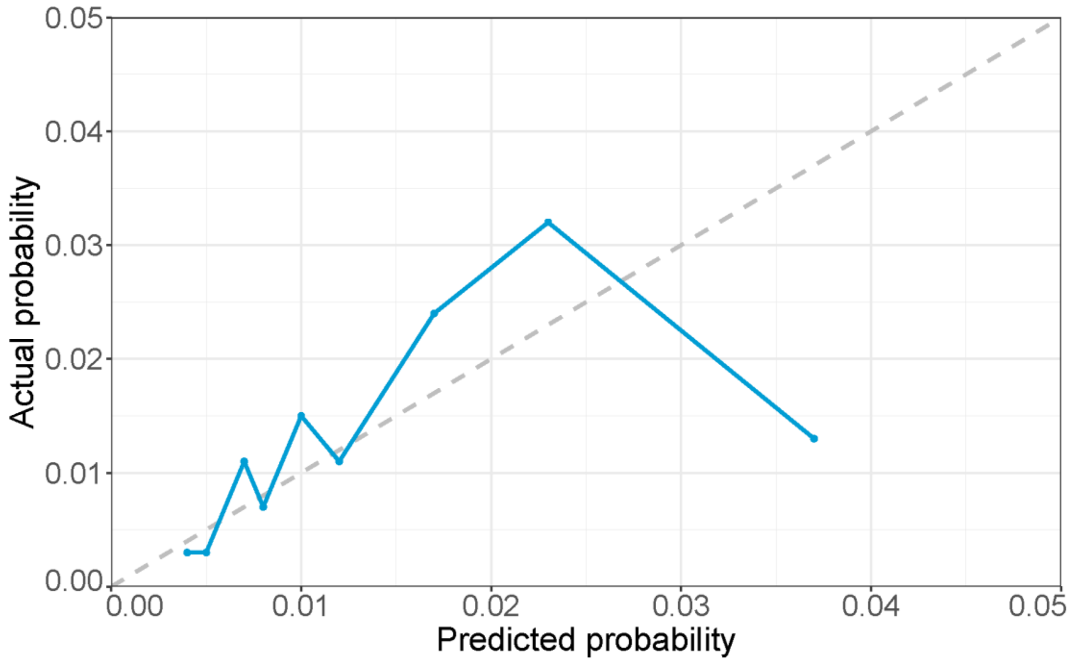

eFigure 3. The calibration plot of Model 2. Model 2, including maternal nutritional condition factors.

© 2023 Jiang H et al. *JAMA Network Open.*

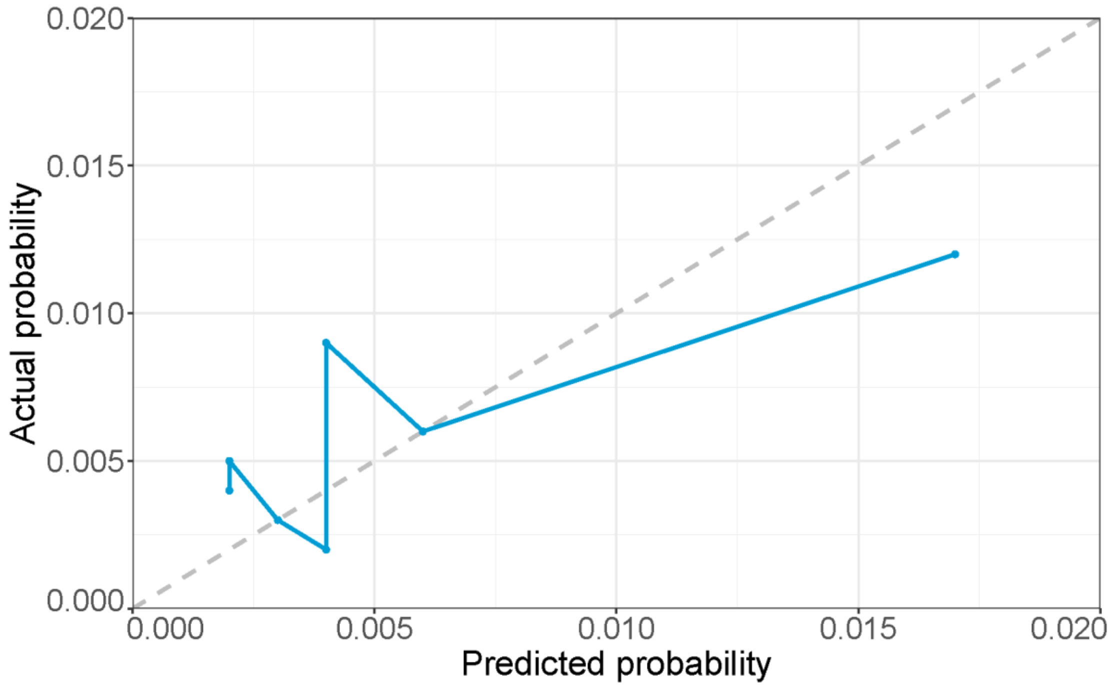

eFigure 4. The calibration plot of Model 3. Model 3, including gestational complications/comorbidities and medication use factors.

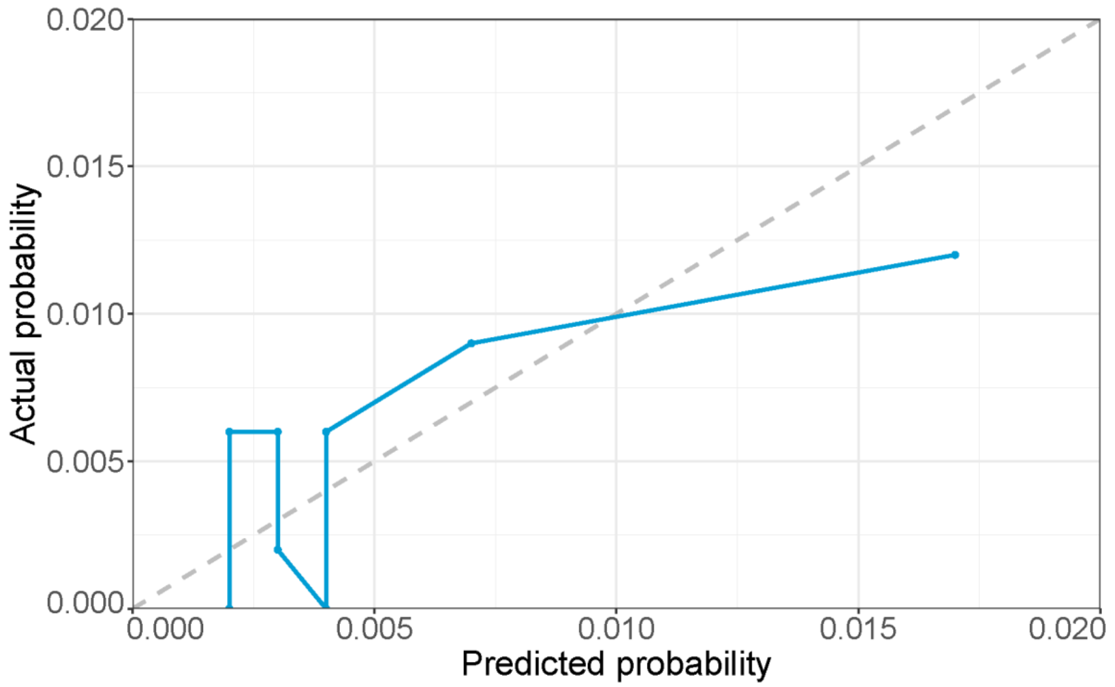

eFigure 5. The calibration plot of Model 4. Model 4, including all prenatal factors.

© 2023 Jiang H et al. *JAMA Network Open.*

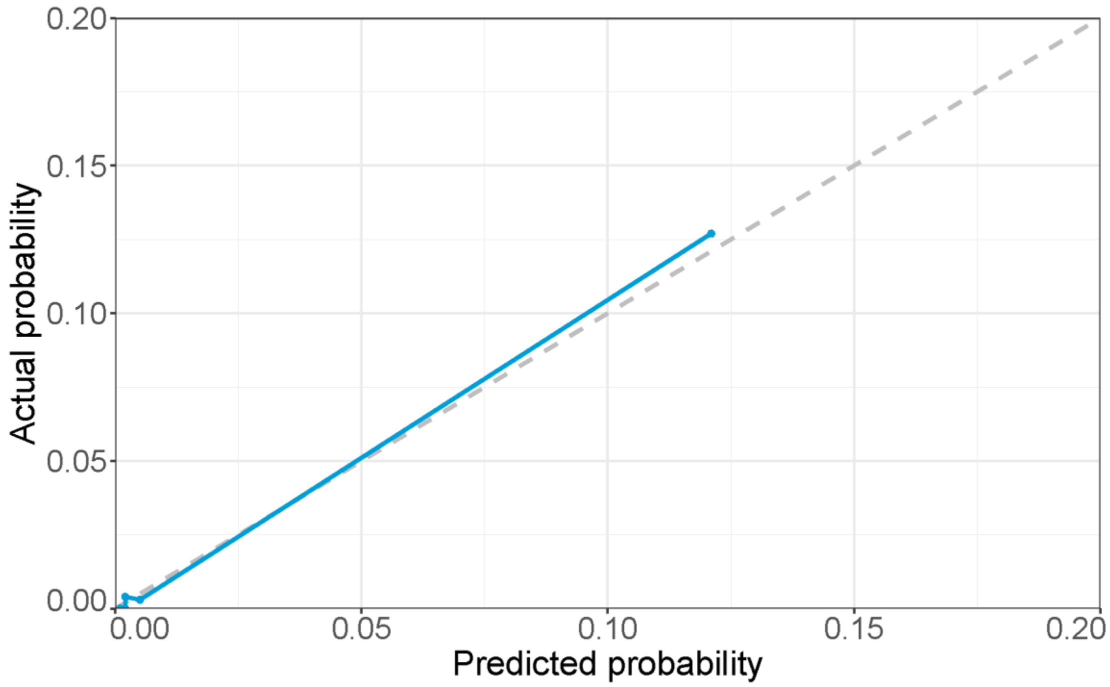

eFigure 6. The calibration plot of Model 5. Model 5, including postnatal factors.

© 2023 Jiang H et al. *JAMA Network Open.*

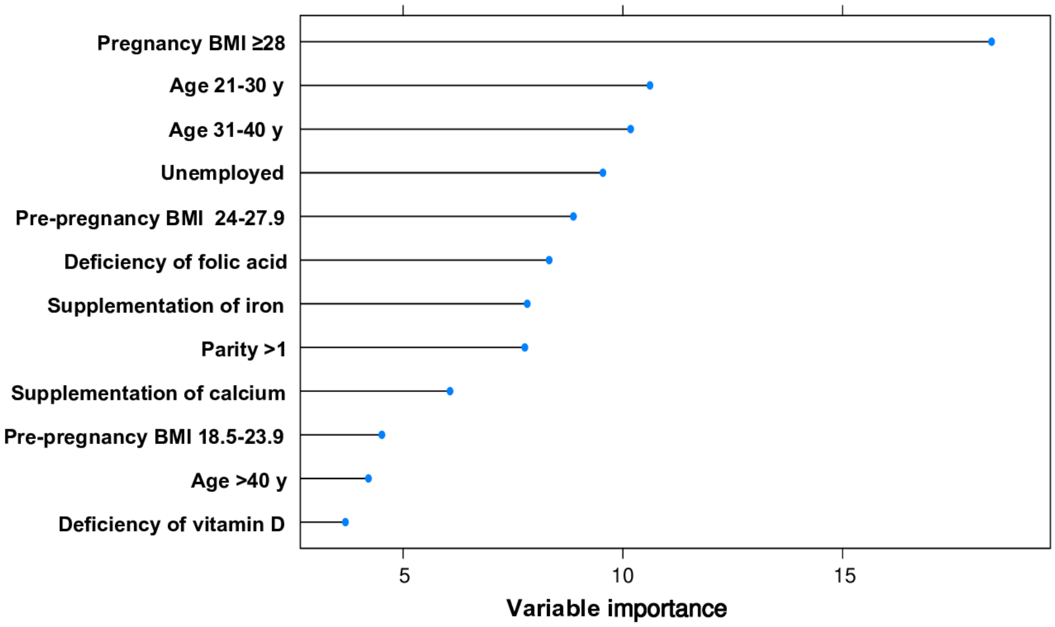

eFigure 7. The variable importance of Model 2 for predicting the risk of MBD in neonates

ANN, artificial neural network; MBD, metabolic bone disease; RDS, respiratory distress syndrome; BMI, body mass index.

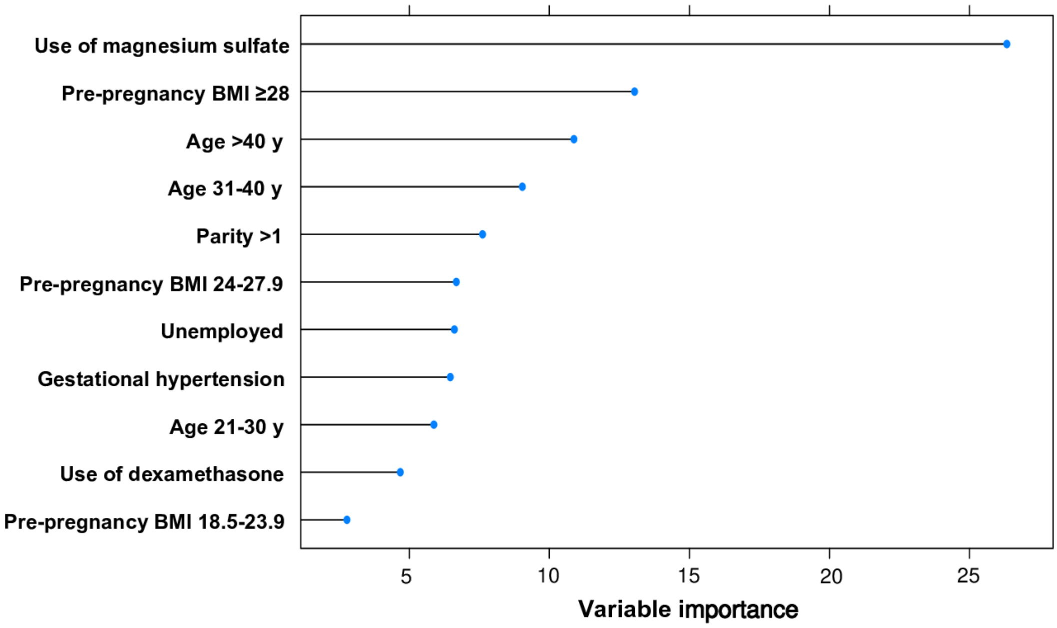

eFigure 8. The variable importance of Model 3 for predicting the risk of MBD in neonates

ANN, artificial neural network; MBD, metabolic bone disease; RDS, respiratory distress syndrome; BMI, body mass index.

© 2023 Jiang H et al. *JAMA Network Open.*

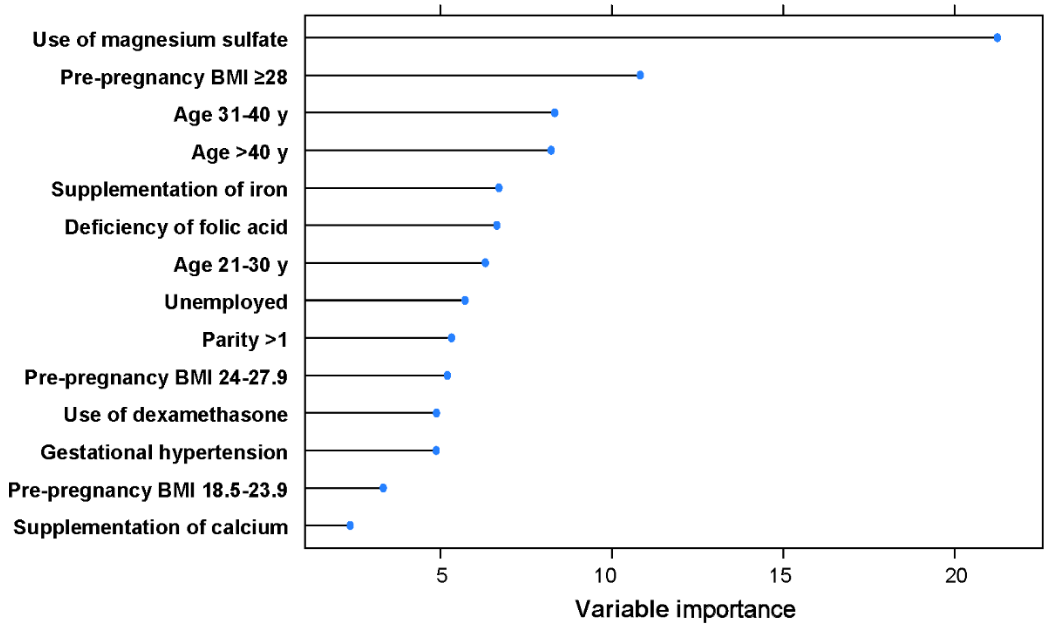

eFigure 9. The variable importance of Model 4 for predicting the risk of MBD in neonates

ANN, artificial neural network; MBD, metabolic bone disease; RDS, respiratory distress syndrome; BMI, body mass index.

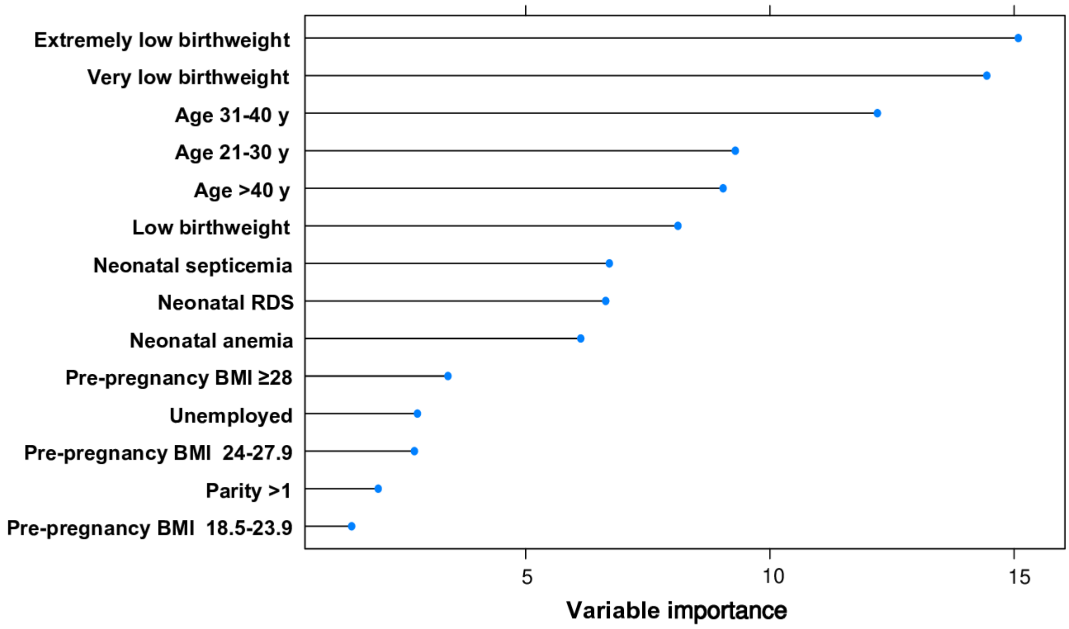

eFigure 10. The variable importance of Model 5 for predicting the risk of MBD in neonates

ANN, artificial neural network; MBD, metabolic bone disease; RDS, respiratory distress syndrome; BMI, body mass index.

© 2023 Jiang H et al. *JAMA Network Open.*

eReference

1. Riley RD, Ensor J, Snell KIE, et al. Calculating the sample size required for developing a clinical prediction model. *BMJ*. 2020;368:m441.

© 2023 Jiang H et al. *JAMA Network Open.*
