# Supplementary material for: Artificial Neural Network Modeling to Predict Neonatal Metabolic Bone Disease in the Prenatal and Postnatal Periods
Source: JAMA Netw Open. 2023 Jan 23;6(1):e2251849. doi: 10.1001/jamanetworkopen.2022.51849 (PMC9871802; doi:10.1001/jamanetworkopen.2022.51849)
Supplement: Supplement 2. — Data Sharing Statement [file jamanetwopen-e2251849-s002.pdf]

Data Sharing Statement

Jiang. Artificial Neural Network Modeling to Predict Neonatal Metabolic Bone Disease in the Prenatal and Postnatal Periods. *JAMA Netw Open*. Published January 23, 2023. doi:10.1001/jamanetworkopen.2022.51849

Data
Data available: Yes
Data types: Deidentified participant data
How to access data: The data will be available upon request after publication at jinghua@tongji.edu.cn
When available: With publication

Supporting Documents
Document types: None

Additional Information
Who can access the data: Researchers whose proposed use of the data has been approved and a data access agreement has been signed.
Types of analyses: For a specified purpose approved by study authors
Mechanisms of data availability: After approval of proposal and with a signed data access agreement
